# Acyl-Hydrazide Derivatives of a Xanthine Carboxylic Congener (XCC) as Selective Antagonists at Human A_2B_ Adenosine Receptors

**DOI:** 10.1002/(sici)1098-2299(199908)47:4<178::aid-ddr4>3.0.co;2-l

**Published:** 1999-08

**Authors:** Yong-Chul Kim, Yishai Karton, Xiao-duo Ji, Neli Melman, Joel Linden, Kenneth A. Jacobson

**Affiliations:** 1 Molecular Recognition Section, Laboratory of Bioorganic Chemistry, National Institute of Diabetes, Digestive and Kidney Diseases, National Institutes of Health, Bethesda, Maryland, USA; 2 Department of Chemistry, Israel Institute of Biological Research, Ness Ziona, Israel; 3 Department of Internal Medicine and Molecular Physiology and Biological Physics, University of Virginia, Health Science Center, Charlottesville, Virginia

**Keywords:** G protein-coupled receptors, radioligand, alkylxanthines, structure–activity relationships, purines, adenylyl cyclase

## Abstract

The structure–activity relationships (SAR) of 8-phenyl-1,3-dipropylxanthine derivatives in binding to recombinant human A_2B_ adenosine receptors were explored, in order to identify selective antagonists. Based on the finding of receptor selectivity in MRS 1204, containing an N-hydroxysuccinimide ester attached through the *p*-position of the 8-phenyl substituent [Jacobson et al. (1999): Drug Dev. Res., 47:45–53], a hydrazide and its more stable imide derivatives were synthesized. The hydrazide of XCC (8-[4-[[[carboxy]methyl]oxy]phenyl]-1,3-dipropylxanthine) was acylated with a variety of mono- and dicarboxylic acids. K_i_ values were determined in the adenosine receptor binding assays. At recombinant human A_2B_ receptors expressed in membranes of HEK-293 cells, antagonist radioligands used were the xanthine ^125^I-ABOPX (^125^I-3-(4-amino-3-iodobenzyl)-8-oxyacetate-1-propyl-xanthine) and the nonxanthine antagonist [^3^H]ZM 241385 ([^3^H]4-(2-[7-amino-2-{furyl}{1,2,4}triazolo{2,3-a}{1,3,5}triazin-5-ylamino-ethyl)phenol). The initial screening utilized rat A_1_/A_2A_ receptors and human A_3_ receptors, and selected compounds were examined at the human A_1_/A_2A_ subtypes. A 1,2-dimethylmaleimide derivative, **14** (MRS 1595), bound to human A_2B_ receptors with a Ki of 19 nM and proved to be selective vs. human A_1_/A_2A_/A_3_ receptors by 160-, 100-, and 35-fold, respectively. Enprofylline (3-propylxanthine) is slightly selective for A_2B_ receptors, suggesting removal of the 1-propyl group; however, combination of the 1-H-3-Pr and 8-phenyl substituents eliminated the selectivity. Other potent and moderately selective A_2B_ antagonists were a tetrahydrophthaloyl derivative **18b** (MRS 1614, K_i_ value 10 nM) and amino acid conjugates of the XCC-hydrazide, i.e., the glutarimide **24b** (MRS 1626, K_i_ value 13 nM), and protected dipeptide **27** (MRS 1615, K_i_ value 11 nM). Drug Dev. Res. 47:178–188, 1999.

## Graphical Abstract

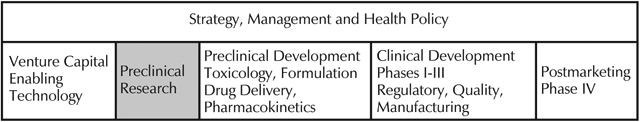

## INTRODUCTION

Adenosine receptors [Linden and Jacobson, 1998] constitute four members of the G protein-coupled receptor superfamily, have structure–function homology to the biogenic amine receptors [Jiang et al., 1997], and are widely distributed in the body. Adenosine is a local modulator in the cardiovascular, renal, and immune systems and in the central nervous system. The A_2B_ adenosine receptor [Daly et al., 1983; see review by Feoktistov and Biaggioni, 1997] is involved in the control of cell growth and gene expression [Neary et al., 1996], vasodilation [Martin et al., 1993], and fluid secretion from intestinal epithelia [Strohmeier et al., 1995].

A selective A_2B_ receptor antagonist may have potential use as an antiasthmatic agent [Feoktistov and Biaggioni, 1997]. A possible role for A_2B_ARs in asthma is consistent with the therapeutic efficacy of enprofylline, **1**, and theophylline, **2**, in treating asthma. In radioligand binding assays, both of these xanthines were confirmed to be effective, although not very potent, antagonists of human A_2B_ARs in the therapeutic dose range [Jacobson et al., 1999]. Furthermore, enprofylline, with a K_i_ value of 7 μM, even appears to be somewhat selective for human A_2B_ARs [Robeva et al., 1996b]. A_2B_ARs are expressed in some mast cells, such as canine BR mastocytoma cells, in which they appear to be responsible for triggering acute Ca^2+^ mobilization and degranulation [Auchampach et al., 1997]. A_2B_ARs also participate in a delayed IL8 release from human HMC-1 mast cells [Feoktistov et al., 1999]. The A_3_AR may also play a role in asthma, since it mediates the degranulation of rat RBL mast-like cells [Ramkumar et al., 1993] and is present in high density in human blood eosinophils [Kohno et al., 1996].

Although adenosine receptor subtype-selective probes are available for the A_1_, A_2A_, and A_3_ adenosine receptors [Jacobson and van Rhee, 1997], very few selective antagonists and agonists are known for the A_2B_ receptor, in part because the absence of radioligand binding assays has precluded a detailed investigation of the SAR at this subtype. MRS 1224, **7b**, a derivative of the triazoloquinazoline, CGS15943, **7a**, was highly potent at the A_2B_ receptor [Kim et al., 1998]. Although selective for the A_2A_ receptor, the triazolotriazine ZM 241385 was also shown to be a potent antagonist at the A_2B_ receptor and useful as a radioligand in cells expressing the recombinant A_2B_ receptor [Ji and Jacobson, 1999]. Alloxazine, **6**, [Brackett and Daly, 1994] has been reported to be approximately one order of magnitude selective as antagonists at the A_2B_ receptor vs. other subtypes. Among xanthines, an 8-phenyl group is associated with increased affinity at A_2B_ receptors. The 8-phenyl analog, **3**, of theophylline, **2**, displayed a 22-fold enhancement of affinity at A_2B_ receptors [Jacobson et al., 1999]. A lead for achieving moderate selectivity (at least 20-fold vs. A_1_, A_2A_, and A_3_ adenosine receptors) have been found in the category of complex 8-phenylxanthine derivatives. 8-[4-[[[Carboxy]methyl]oxy]phenyl]-1,3-dipropylxanthine (XCC), **4a**, and its ethyl ester, **4b**, displayed high affinity for the A_2B_ receptor. Moreover, MRS 1204 (N-hydroxysuccinimide ester of XCC), **4d**, displayed moderate selectivity (at approximately 20-fold for human A_2B_ receptors [Jacobson et al., 1999] vs. A_1_, A_2A_, and A_3_ adenosine receptors).

As an approach to finding selective antagonists for the A_2B_ receptor, we synthesized novel 8-phenyl-1,3-dialkylxanthines related structurally to **4d**, in most of which the active ester bond has been replaced by a more stable acyl-hydrazide bond, and screened them for receptor affinity and selectivity in binding to the recombinant human A_2B_ receptor and other adenosine receptor subtypes. In order to identify potent adenosine receptor subtype-selective antagonists, in this study we utilized radioligand binding assays based on the use of membranes derived from HEK-293 cells that overexpress recombinant human A_2B_ARs.

## MATERIALS AND METHODS

### Materials

The starting compounds, **4c** and **4b**, were prepared according to Jacobson et al. [1985]. NECA, XAC, and 2-chloroadenosine were purchased from Research Biochemicals International (Natick, MA). All reagents were obtained from Aldrich (Milwaukee, WI) and Sigma (St. Louis, MO).

### Synthesis

Proton nuclear magnetic resonance spectroscopy was performed on a Varian GEMINI-300 spectrometer and spectra were taken in DMSO-*d*_6_ or CDCl_3_. Unless noted, chemical shifts are expressed as ppm downfield from tetramethylsilane, or relative ppm from DMSO (2.5 ppm). Chemical-ionization (CI) mass spectrometry was performed with a Finnigan 4600 mass spectrometer, and Electron-impact (EI) mass spectrometry with a VG7070F mass spectrometer at 6 kV. FAB (fast atom bombardment) mass spectrometry was performed with a JEOL SX102 spectrometer using 6-kV Xe atoms. All xanthine derivatives tested in binding assays were shown to be homogeneous by TLC (MK6F silica, 0.25 mm, glass-backed; Whatman Inc., Clifton, NJ). NMR and mass spectra were shown to be consistent with the assigned structure.

### General Procedure for the Preparation of Xanthine Hydrazide Derivatives

#### Carboxyalkyl amide derivatives

A mixture of **4c** (10 mg, 0.025 mmol) and two equivalents of anhydride were stirred in 1 mL of DMF for 6–24 h. The reaction mixture was concentrated to dryness and the residue was purified on preparative TLC (CHCl_3_: MeOH = 10:1) to give the corresponding carboxyalkylamide derivative as a white solid with 40–70% yield (compounds **4e**, **9**, **18a**, **19a** and **20a**).

#### Cyclic imide derivatives

A mixture of **4c** (10 mg, 0.025 mmol), 1.5–2.0 equivalents of anhydride, and one equivalent of DIPEA were stirred in 1 mL of DMF at room temperature. When the starting material **4c** disappeared as judged by TLC, a mixture of 2–3 equivalents of HOBt, EDAC, and DIPEA dissolved in 0.5 mL of DMF was added and the mixture was stirred at room temperature or at 50°C for 6–24 h. The reaction mixture was concentrated to dryness and the residue was purified on preparative TLC (CHCl_3_: MeOH = 10:1) to give the cyclic imide derivative as a white solid, 40–70% yield (compounds **10**, **11**, **12**, **13**, **14**, **15**, **16**, **17**, **18b**, **19b**, **20b**, **21**, **22**, **23**).

#### Coupling with activated N-protected amino acids

A mixture of **4c** (10 mg, 0.025 mmol), 1.5–2.0 equivalents of activated (hydroxy-succinimide or 4-nitrophenyl ester) N-protected amino acid and one equivalent of DIPEA and DMAP was stirred in 1 mL of DMF at 25–50°C for 8–24 h. The reaction mixture was concentrated to dryness and the residue was purified on preparative TLC (CHCl_3_: MeOH = 10:1) to give the product as a white solid, 40–70% yield (compounds **25**, **26**, and **27**).

##### 8-[4-[(Carboxymethyl)oxy]phenyl]-1,3-di-(*n*-propyl)xanthine N-Acetylhydrazide (4e).

^1^H NMR (DMSO-d_6_). 0.89 (2t, 6H, *J* = 7.8 Hz, 2x-CH_3_), 1.58 and 1.74 (2m, 4H, 2x-CH_2_-), 1.88 (s, 3H, CH_3_CO-), 3.87 and 4.02 (2t, 4H, *J* = 6.8 Hz, 2x-NCH_2_-), 4.68 (s, 2H, -OCH_2_-), 7.11 (d, 2H, *J* = 8.8 Hz, Ar), 8.08 (d, 2H, *J* = 8.8 Hz, Ar); MS-FAB (M + H^+^) 443.

##### 8-[4-[(Carboxymethyl)oxy]phenyl]-1,3-di-(*n*-propyl)xanthine *N*-[(3-Carboxy)-*n*-propionyl]hydrazide (9).

^1^H NMR (DMSO-d_6_). 0.89 (2t, 6H, *J* = 7.8 Hz, 2x-CH_3_), 1.58 and 1.74 (2m, 4H, 2x-CH_2_-), 2.43 (m, 4H, -COCH_2_CH_2_CO-), 3.87 and 4.02 (2t, 4H, *J* = 6.8 Hz, 2x-NCH_2_-), 4.67 (s, 2H, -OCH_2_-), 7.11 (d, 2H, *J* = 8.8 Hz, Ar), 8.08 (d, 2H, *J* = 8.8 Hz, Ar); MS-FAB (M + H^+^) 501.

##### 8-[4-[(Carboxymethyl)oxy]phenyl]-1,3-di-(n-propyl)xanthine N,N-Succinylhydrazide (10).

^1^H NMR (DMSO-d_6_). 0.89 (2t, 6H, *J* = 7.8 Hz, 2x-CH_3_), 1.59 and 1.73 (2m, 4H, 2x-CH_2_-), 2.81 (s, 4H, CH_2_CH_2_), 3.87 and 4.03 (2t, 4H, *J* = 6.8 Hz, 2x-NCH_2_-), 4.85 (s, 2H, -OCH_2_-), 7.15 (d, 2H, *J* = 8.8 Hz, Ar), 8.10 (d, 2H, *J* = 8.8 Hz, Ar); MS-FAB (M + H^+^) 483.

##### 8-[4-[(Carboxymethyl)oxy]phenyl]-1,3-di-(*n*-propyl)xanthine *N*,*N*-[((2S)-Trifluoroacetamido)- succinyl]hydrazide (11).

^1^H NMR (DMSO-d_6_). 0.89 (2t, 6H, *J* = 7.8 Hz, 2x-CH_3_), 1.58 and 1.74 (2m, 4H, 2x-CH_2_-), 2.70–2.90 (m, 2H, -CH_2_-), 3.81 and 3.98 (2t, 4H, *J* = 6.8 Hz, 2x-NCH_2_-), 4.69 (s, 2H, -OCH_2_-), 4.95 (s, 1H, -CH-), 7.15 (d, 2H, *J* = 8.8 Hz, Ar), 8.10 (d, 2H, *J* = 8.8 Hz, Ar); MS-FAB (M + H^+^) 594.

##### 8-[4-[(Carboxymethyl)oxy]phenyl]-1,3-di-(*n*-propyl)xanthine *N,N*-[(2-Phenyl)glutaryl]hydrazide (12).

^1^H NMR (CDCl_3_). 1.05 (2t, 6H, *J* = 7.8 Hz, 2x-CH_3_), 1.75 and 1.90 (2m, 4H, 2x-CH_2_-), 2.3–2.5 and 2.8–3.1 (m, 5H, -CH- and 2x-CH_2_-), 4.04 and 4.12 (2t, 4H, *J* = 6.8 Hz, 2x-NCH_2_-), 4.70–4.90 (m, 2H, -OCH_2_-), 6.6 (d, 2H, *J* = 8.8 Hz, Ar), 7.08 (m, 2H, -Ph), 7.43 (m, 5H, -Ph and Ar); MS-FAB (M + H^+^) 573.

##### 8-[4-[(Carboxymethyl)oxy]phenyl]-1,3-di-(*n*-propyl)xanthine *N,N*-Citraconylhydrazide (13).

^1^H NMR (DMSO-d_6_). 0.89 (2t, 6H, *J* = 7.8 Hz, 2x-CH_3_), 1.59 and 1.73 (2m, 4H, 2x-CH_2_-), 2.07 (s, 3H, CH_3_), 3.87 and 4.03 (2t, 4H, *J* = 6.8 Hz, 2x-NCH_2_-), 4.86 (s, 2H, -OCH_2_-), 6.83 (s, 1H, =CH-), 7.15 (d, 2H, *J* = 8.8 Hz, Ar), 8.10 (d, 2H, *J* = 8.8 Hz, Ar); MS-FAB (M + H^+^) 495.

##### 8-[4-[(Carboxymethyl)oxy]phenyl]-1,3-di-(*n*-propyl)xanthine *N,N*-[(1,2-Dimethyl)maleyl]hydrazide (14).

^1^H NMR (DMSO-d_6_). 0.89 (2t, 6H, *J* = 7.8 Hz, 2x-CH_3_), 1.58 and 1.74 (2m, 4H, 2x-CH_2_-), 1.97 (s, 6H, 2x-CH_3_), 3.87 and 4.03 (2t, 4H, *J* = 6.8 Hz, 2x-NCH_2_-), 4.86 (s, 2H, -OCH_2_-), 7.14 (d, 2H, *J* = 8.8 Hz, Ar), 8.10 (d, 2H, *J* = 8.8 Hz, Ar); MS-FAB (M + H^+^) 509.

##### 8-[4-[(Carboxymethyl)oxy]phenyl]-1H-3-(*n*-propyl)xanthine *N,N*-[(1,2-Dimethyl)maleyl]hydrazide (15).

^1^H NMR (DMSO-d_6_). 0.91 (t, 3H, *J* = 7.8 Hz, 2x-CH_3_), 1.73 (m, 2H, -CH_2_-), 1.97 (s, 6H, 2x-CH_3_), 3.96 (t, 2H, *J* = 6.8 Hz, 2x-NCH_2_-), 4.85 (s, 2H, -OCH_2_-), 7.14 (d, 2H, *J* = 8.8 Hz, Ar), 8.09 (d, 2H, *J* = 8.8 Hz, Ar); MS-EI (M^+^) 509, calculated for C_22_H_22_N_6_O_6_ 466.1601; found 466.1580.

##### 8-[4-[(Carboxymethyl)oxy]phenyl]-1,3-di-(*n*-propyl)xanthine *N,N*-[(2-Phenyl)maleyl]hydrazide (16).

^1^H NMR (DMSO-d_6_). 0.89 (2t, 6H, *J* = 7.8 Hz, 2x-CH_3_), 1.59 and 1.73 (2m, 4H, 2x-CH_2_-), 3.87 and 4.03 (2t, 4H, *J* = 6.8 Hz, 2x-NCH_2_-), 4.91 (s, 2H, -OCH_2_-), 7.15 (d, 2H, *J* = 8.8 Hz, Ar), 7.51 (s, 1H, =CH-), 7.55–7.57 (m, 3H, -Ph), 8.04–8.06 (m, 2H, -Ph), 8.11 (d, 2H, *J* = 8.8 Hz, Ar); MS-FAB (M + H^+^) 557.

##### 8-[4-[(Carboxymethyl)oxy]phenyl]-1,3-di-(*n*-propyl)xanthine *N,N*-[(1,2-Diphenyl)maleyl]hydrazide (17).

^1^H NMR (DMSO-d_6_). 0.89 (2t, 6H, *J* = 7.8 Hz, 2x-CH_3_), 1.59 and 1.73 (2m, 4H, 2x-CH_2_-), 3.87 and 4.03 (2t, 4H, *J* = 6.8 Hz, 2x-NCH_2_-), 4.94 (s, 2H, -OCH_2_-), 7.15 (d, 2H, *J* = 8.8 Hz, Ar), 7.45 (bs, 10H, 2x-Ph), 8.10 (d, 2H, *J* = 8.8 Hz, Ar); MS-FAB (M + H^+^) 633.

##### 8-[4-[(Carboxymethyl)oxy]phenyl]-1,3-di-(*n*-propyl)xanthine *N*-[2-((1-Carboxy)-cis-4-cyclohexene)-carbonyl]hydrazide (18a).

^1^H NMR (DMSO-d_6_). 0.89 (2t, 6H, *J* = 7.8 Hz, 2x-CH_3_), 1.58 and 1.74 (2m, 4H, 2x-CH_2_-), 2.30–2.50 (m, 4H, 2x-CH_2_-), 2.80–2.95 (m, 2H, 2x-CH-), 3.83 and 3.90 (2t, 4H, *J* = 6.8 Hz, 2x-NCH_2_-), 4.66 (s, 2H, -OCH_2_-), 5.63 (s, 2H, 2 x =CH-), 7.09 (d, 2H, *J* = 8.8 Hz, Ar), 8.06 (d, 2H, *J* = 8.8 Hz, Ar); MS-FAB (M + H^+^) 553.

##### 8-[4-[(Carboxymethyl)oxy]phenyl]-1,3-di-*(n*-propyl)xanthine *N,N*-(*cis*-1,2,3,6-Tetrahydrophthaloyl)-hydrazide (18b).

^1^H NMR (DMSO-d_6_). 0.89 (2t, 6H, *J* = 7.8 Hz, 2x-CH_3_), 1.58 and 1.74 (2m, 4H, 2x-CH_2_-), 2.20–2.50 (m, 4H, 2x-CH_2_-), 3.56 (m, 2H, 2x-CH-), 3.83 and 3.90 (2t, 4H, *J* = 6.8 Hz, 2x-NCH_2_-), 4.66 (s, 2H, -OCH_2_-), 5.89 (s, 2H, 2 x = CH-), 7.09 (d, 2H, *J* = 8.8 Hz, Ar), 8.06 (d, 2H, *J* = 8.8 Hz, Ar); MS-FAB (M + H^+^) 535.

##### 8-[4-[(Carboxymethyl)oxy]phenyl]-1,3-di-(*n*-propyl)xanthine *N*-[2-((1-Carboxy)-1-cyclopentene)-carbonyl]hydrazide (19a).

^1^H NMR (DMSO-d_6_). 0.89 (2t, 6H, *J* = 7.8 Hz, 2x-CH_3_), 1.58 and 1.74 (2m, 4H, 2x-CH_2_-), 1.87 (m, 2H, -CH_2_-), 2.70 (m, 4H, 2x-CH_2_-), 3.83 and 3.90 (2t, 4H, *J* = 6.8 Hz, 2x-NCH_2_-), 4.71 (s, 2H, -OCH_2_-), 7.09 (d, 2H, *J* = 8.8 Hz, Ar), 8.06 (d, 2H, *J* = 8.8 Hz, Ar); MS-FAB (M + H^+^) 539.

##### 8-[4-[(Carboxymethyl)oxy]phenyl]-1,3-di-(*n*-propyl)xanthine *N,N*-(1-Cyclopentene-1,2-dicarbonyl)-hydrazide (19b).

^1^H NMR (DMSO-d_6_). 0.89 (2t, 6H, *J* = 7.8 Hz, 2x-CH_3_), 1.58 and 1.74 (2m, 4H, 2x-CH_2_-), 2.40 (m, 2H, -CH_2_-), 2.67(4H, m, 2x-CH_2_-), 3.81 and 3.98 (2t, 4H, *J* = 6.8 Hz, 2x-NCH_2_-), 4.85 (s, 2H, -OCH_2_-), 7.15 (d, 2H, *J* = 8.8 Hz, Ar), 8.1 (d, 2H, *J* = 8.8 Hz, Ar); MS-FAB (M + H^+^) 521.

##### 8-[4-[(Carboxymethyl)oxy]phenyl]-1,3-di-(*n*-propyl)xanthine *N*-[2-((1-Carboxy)-1-cyclohexene)-carbonyl]hydrazide (20a).

^1^H NMR (DMSO-d_6_). 0.89 (2t, 6H, *J* = 7.8 Hz, 2x-CH_3_), 1.59 (m, 6H, 3x-CH_2_-), 1.74 (m, 2H, -CH_2_-), 2.27 (m, 4H, 2x-CH_2_-), 3.87 and 4.02 (2t, 4H, *J* = 6.8 Hz, 2x-NCH_2_-), 4.68 (s, 2H, -OCH_2_-), 7.09 (d, 2H, *J* = 8.8 Hz, Ar), 8.06 (d, 2H, *J* = 8.8 Hz, Ar); MS-FAB (M + H^+^) 553.

##### 8-[4-[(Carboxymethyl)oxy]phenyl]-1,3-di-(*n*-propyl)xanthine *N,N*-(3,4,5,6-Tetrahydrophthaloyl)-hydrazide (20b).

^1^H NMR (DMSO-d_6_). 0.89 (2t, 6H, *J* = 7.8 Hz, 2x-CH_3_), 1.58 (m, 2H, -CH_2_-), 1.72 (m, 6H, 3x-CH_2_-), 2.30 (m, 4H, 2x-CH_2_-) 3.83 and 3.90 (2t, 4H, *J* = 6.8 Hz, 2x-NCH_2_-), 4.86 (s, 2H, -OCH_2_-), 7.15 (d, 2H, *J* = 8.8 Hz, Ar), 8.12 (d, 2H, *J* = 8.8 Hz, Ar); MS-FAB (M + H^+^) 535.

##### 8-[4-[(Carboxymethyl)oxy]phenyl]-1,3-di-(*n*-propyl)xanthine *N,N*-Phthaloylhydrazide (21).

^1^H NMR (DMSO-d_6_). 0.89 (2t, 6H, *J* = 7.8 Hz, 2x-CH_3_), 1.58 and 1.74 (2m, 4H, 2x-CH_2_-), 3.87 and 4.02 (2t, 4H, *J* = 6.8 Hz, 2x-NCH_2_-), 4.75 (s, 2H, -OCH_2_-), 7.14 (d, 2H, *J* = 8.8 Hz, Ar), 7.57 (m, 4H, Ar), 8.09 (d, 2H, *J* = 8.8 Hz, Ar); MS-FAB (M + H^+^) 531.

##### 8-[4-[(Carboxymethyl)oxy]phenyl]-1,3-di-(*n*-propyl)xanthine *N,N*-Glutarylhydrazide (22).

^1^H NMR (CDCl_3_). 1.05 (2t, 6H, *J* = 7.8 Hz, 2x-CH_3_), 1.75 and 1.90 (2m, 4H, 2x-CH_2_-), 2.10–2.30 (m, 2H, -CH_2_-), 2.80–3.10 (m, 4H, 2x-CH_2_-), 4.05 and 4.16 (2t, 4H, *J* = 6.8 Hz, 2x-NCH_2_-), 4.80 (s, 2H, -OCH_2_-), 6.75 (d, 2H, *J* = 8.8 Hz, Ar), 7.70 (d, 2H, *J* = 8.8 Hz, Ar); MS-FAB (M + H^+^) 497.

##### 8-[4-[(Carboxymethyl)oxy]phenyl]-1,3-di-(*n*-propyl)xanthine *N,N*-(3-Hydroxy)glutarylhydrazide (23).

^1^H NMR (DMSO-d_6_). 0.89 (2t, 6H, *J* = 7.8 Hz, 2x-CH_3_), 1.59 and 1.73 (2m, 4H, 2x-CH_2_-), 2.70–3.10 (m, 4H, 2x-CH_2_-), 3.87 and 4.03 (2t, 4H, *J* = 6.8 Hz, -NCH_2_-), 4.21 (bs, 1H, -C*H*OH-), 4.77 (s, 2H, -OCH_2_-), 7.15 (d, 2H, *J* = 8.8 Hz, Ar), 8.1 (d, 2H, *J* = 8.8 Hz, Ar); MS-FAB (M + H^+^) 513.

##### 8-[4-[(Carboxymethyl)oxy]phenyl]-1,3-di-(*n*-propyl)xanthine *N*-[(4-Carboxy-(2S)-Trifluoroacetamido)-*n*-butanoyl]hydrazide (24a).

A mixture of **4c** (10 mg, 0.025 mmol), 7.6 mg of l-N-Boc-glutamic acid 5-*tert*-butyl ester (0.025 mmole), 7 mg of HOBt (0.05 mmole), 19 mg of DIPEA (0.15 mmole), and 15 mg of EDAC (0.078 mmole) in 1 mL of dry DMF was stirred for 8 h at 25°C. DMF was removed by nitrogen stream and the residue was washed with 1 mL of 1 M NaHCO_3_ solution and dried overnight. The crude product was suspended in 0.5 mL of CHCl_3_ and 0.5 mL of TFA was added. After 30 min stirring at 25°C, the mixture was concentrated to dryness and dried under high vacuum. The residue was dissolved in 0.5 mL of TFAA and the solution was stirred for 30 min at 25°C. The reaction mixture was concentrated to dryness and the residue was purified on preparative TLC (CHCl_3_: MeOH = 10:1) to give 6 mg of **24a** as a white solid (yield 40%). ^1^H NMR (DMSO-d_6_). 0.89 (2t, 6H, *J* = 7.8 Hz, 2x-CH_3_), 1.59 and 1.73 (2m, 4H, -CH_2_-), 1.90–2.30 (m, 4H, 2x-CH_2_-), 3.87 and 4.02 (2t, 4H, *J* = 6.8 Hz, 2x-NCH_2_-), 4.12 (m, 1H, -CH-), 4.68 (s, 2H, -OCH_2_-), 7.08 (d, 2H, *J* = 8.8 Hz, Ar), 8.06 (d, 2H, *J* = 8.8 Hz, Ar); MS-FAB (M+ H^+^) 626.

##### 8-[4-[(Carboxymethyl)oxy]phenyl]-1,3-di-(*n*-propyl)xanthine *N,N*-((2S)-Trifluoroacetamido)-glutaryl]hydrazide (24b).

A mixture of **24a** (10 mg, 0.016 mmol), 7 mg of HOBt (0.05 mmole), 19 mg of DIPEA (0.15 mmole), and 15 mg of EDAC (0.078 mmole) in 1 mL of dry DMF was stirred overnight at 25°C. The reaction mixture was concentrated to dryness and the residue was purified on preparative TLC (CHCl_3_:MeOH=10:1) to give 5 mg of **24b** as a white solid (yield 53%). ^1^H NMR (DMSO-d_6_). 0.89 (2t, 6H, *J* = 7.8 Hz, 2x-CH_3_), 1.59 and 1.73 (2m, 4H, 2x-CH_2_-), 1.90–2.30 (m, 4H, 2x-CH_2_-), 3.87 and 4.02 (2t, 4H, *J* = 6.8 Hz, 2x-NCH_2_-), 4.81 (s, 2H, -OCH_2_-), 4.18 (m, 1H, -CH-), 7.15 (d, 2H, *J* = 8.8 Hz, Ar), 8.1 (d, 2H, *J* = 8.8 Hz, Ar); MS-FAB (M + H^+^) 608.

##### 8-[4-[(Carboxymethyl)oxy]phenyl]-1,3-di-(*n*-propyl)xanthine *N*-(*N*-*tert*-Butoxycarbonyl-l-leucinyl)-hydrazide (25).

^1^H NMR (DMSO-d_6_). 0.89 (m, 13H, 2x-CH_3_ and (CH_3_)_2_CH-), 1.35 (s, 9H, Boc), 1.42 (m, 2H, -CH_2_-), 1.58 and 1.74 (2m, 4H, 2x-CH_2_-), 3.85 and 4.0 (2t, 4H, *J* = 6.8 Hz, 2x-NCH_2_-), 4.12 (m, 1H, -CH-), 4.64 (s, 2H, -OCH_2_-), 7.06 (d, 2H, *J* = 8.8 Hz, Ar), 8.05 (d, 2H, *J* = 8.8 Hz, Ar); MS-FAB (M + H^+^) 614.

##### 8-[4-[(Carboxymethyl)oxy]phenyl]-1,3-di-(*n-*propyl)xanthine *N*-(*N*-*tert*-Butoxycarbonyl-l-methionyl)-hydrazide (26).

^1^H NMR (DMSO-d_6_). 0.89 (2t, 6H, *J* = 7.8 Hz, 2x-CH_3_), 1.25 (m, 2H, -CH_2_-), 1.37 (s, 9H, Boc), 1.58 and 1.74 (2m, 4H, 2x-CH_2_-), 1.88 (m, 2H, -CH_2_-), 2.03 (s, 3H, -SCH_3_), 3.81 and 3.98 (2t, 4H, *J* = 6.8 Hz, 2x-NCH_2_-), 4.15 (m, 1H, -CH-), 4.68 (s, 2H, -OCH_2_-), 7.03 (d, 2H, *J* = 8.8 Hz, Ar), 8.03 (d, 2H, *J* = 8.8 Hz, Ar); MS-FAB (M + H^+^) 632.

##### 8-[4-[(Carboxymethyl)oxy]phenyl]-1,3-di-(*n-*propyl)xanthine *N*-(*N*-Benzyloxycarbonyl-glycylglycinyl)hydrazide (27).

^1^H NMR (DMSO-d_6_). 0.89 (2t, 6H, J = 7.8 Hz, 2x-CH_3_), 1.58 and 1.74 (2m, 4H, 2x-CH_2_-), 3.67 (m, 1H, -CH_2_- in glycine), 3.81 (m, 3H, -NCH_2_- and -CH_2_- in glycine), 3.98 (t, 2H, *J* = 6.8 Hz, -NCH_2_-), 4.64 (s, 2H, -OCH_2_-), 5.03(s, 2H, -OC*H*_2_-Ph), 7.03 (d, 2H, *J* = 8.8 Hz, Ar), 7.3–7.5 (m, 5H, -Ph), 8.03 (d, 2H, *J* = 8.8 Hz, Ar); MS-FAB (M + H^+^) 649.

##### 8-[4-[(Carboxymethyl)oxy]phenyl]-1*H*-3-(*n-*propyl)xanthine methyl ester (36).

To a suspension of 3.2 g of **32** [Papesch and Schroeder, 1951] (18.9 mmole), 1.5 mL of glacial acetic acid and 3.4 mL of 6 N HCl in 50 mL of water was added dropwise to a solution of 1.38 g of sodium nitrite (20 mmole) in 5 mL of water at 0°C. The mixture was stirred for 1 h and the pink precipitate was collected by filtration to give 3.17 g of **33** (yield 78%). ^1^H NMR (DMSO-d_6_) 0.87 (t, 3H, *J* = 7.8 Hz, -CH_3_), 1.51 (m, 2H, -CH_2_-), 3.72 (t, 2H, *J* = 6.8 Hz, -NCH_2_-), 9.12 (s, 1H, -NH_2_). 0.086 g of **33** (0.4 mmole) was hydrogenated with 10% Pd/C in 5 mL of MeOH under H_2_ atmosphere (1 atm) at 25°C until the pink color disappeared (30 min). After the removal of the balloon of H_2_, 5 mL of DMF was added and the mixture was stirred for 10 min and filtered through a Celite bed. To the solution of crude **34** was added 0.078 g of methyl 4-formylphenyloxyacetate (0.4 mmole) and 0.5 mL of acetic acid. The mixture was heated at 50°C for 30 min, evaporated under reduced pressure, and suspended with 20 mL of ether. The yellow precipitate (mixture of **35** and **36**) was collected by filtration, dissolved in 5 mL of DMF, and treated with 1 mL of aqueous solution of 0.085 g of sodium periodate (0.4 mmole) for 2 h. After evaporation, the product was purified by crystallization in MeOH/H_2_O to give 0.048 g of **36** (yield 34%). ^1^H NMR (DMSO-d_6_). 0.90 (t, 3H, *J* = 7.8 Hz, -CH_3_), 1.72 (m, 2H, -CH_2_-), 3.71 (s, 3H, -OCH_3_), 3.95 (t, 2H, *J* = 6.8 Hz, -NCH_2_-), 4.89 (s, 2H, -OCH_2_-), 7.08 (d, 2H, *J* = 8.8 Hz, Ar), 8.05 (d, 2H, *J* = 8.8 Hz, Ar), 11.07 (s, 1H, -NH); MS-EI (M^+^) 358, calculated for C_17_H_18_N_4_O_5_ 358.1277; found 358.1269.

##### 8-[4-[(Carboxymethyl)oxy]phenyl]-1*H*-3-(*n-*propyl)xanthine Hydrazide (37).

A solution of 0.05 g of **36** (0.14 mmole) and 0.5 mL of hydrazine anhydrous in 2 mL of dry DMF was heated overnight at 50°C. After evaporation, the residue was suspended in MeOH and the white precipitate was collected by filtration to give 0.025 g of **37** (yield 50%). m.p. = 267°C; ^1^H NMR (DMSO-d_6_). 0.90 (t, 3H, *J* = 7.8 Hz, -CH_3_), 1.72 (m, 2H, -CH_2_-), 3.71 (s, 3H, -OCH_3_), 3.95 (t, 2H, *J* = 6.8 Hz, -NCH_2_-), 4.34 (bs, 2H, NH_2_), 4.56 (s, 2H, -OCH_2_-), 7.08 (d, 2H, *J* = 8.8 Hz, Ar), 8.05 (d, 2H, *J* = 8.8 Hz, Ar), 9.39 (s, 1H, -NH); MS-EI (M^+^) 358, calculated for C_16_H_18_N_6_O_4_ 358.1389; found 358.1389.

### Pharmacology

The human A_2B_ receptor cDNA was subcloned into the expression plasmid pDoubleTrouble [Robeva et al., 1996a]. The plasmid was amplified in competent JM109 cells and plasmid DNA isolated using Wizard Megaprep columns (Promega Corp., Madison, WI). A_2B_ adenosine receptors were introduced into HEK-293 cells by means of Lipofectin [Felgner et al., 1987].

#### Cell culture

Transfected HEK cells were grown under 5% CO_2_/95% O_2_ humidified atmosphere at a temperature of 37°C. Colonies were selected by growth of cells in 0.6 mg/mL G418. Transfected cells were maintained in DMEM supplemented with Hams F12 nutrient mixture (1/1), 10% newborn calf serum, 2 mM glutamine, and containing 50 IU/mL penicillin, 50 μg/mL streptomycin, and 0.2 mg/mL Geneticin (G418, Boehringer Mannheim, Indianapolis, IN). Cells were cultured in 10 cm diameter round plates and subcultured when grown confluent (approximately after 72 h).

#### Radioligand binding studies

Confluent monolayers of HEK-A_2B_ cells were washed with PBS followed by ice-cold Buffer A (10 mM HEPES, 10 mM EDTA, pH 7.4) with protease inhibitors (10 mg/mL benzamidine, 100 mM phenylmethanesulfonyl fluoride, and 2 mg/mL of each aprotinin, pepstatin, and leupeptin). The cells were homogenized in a Polytron (Brinkmann) for 20 sec, centrifuged at 30,000*g*, and the pellets washed twice with buffer HE (10 mM HEPES, 1 mM EDTA, pH 7.4 with protease inhibitors). The final pellet was resuspended in buffer HE, supplemented with 10% sucrose and frozen in aliquots at −80°C. For binding assays, membranes were thawed and diluted 5–10-fold with HE to a final protein concentration of approximately 1 mg/mL. To determine protein concentrations, membranes, and bovine serum albumin standards were dissolved in 0.2% NaOH/0.01% SDS and protein determined using fluorescamine fluorescence [Stowell et al., 1978]. Saturation binding assays for human A_2B_ adenosine receptors were performed with [^125^I-]ABOPX (2,200 Ci/mmol). To prepare [^125^I-]ABOPX, 10 mL of 1 mM ABOPX in methanol/1 M NaOH (20:1) was added to 50 mL of 100 mM phosphate buffer, pH 7.3. One or 2 mCi of Na^125^I was added, followed by 10 mL of 1 mg/mL chloramine-T in water. After incubating for 20 min at room temperature, 50 mL of 10 mg/mL Na-metabisulfite in water was added the quench the reaction. The reaction products were applied to a C18 HPLC column using 4 mM phosphate, pH 6.0/methanol. After 5 min in 35% methanol, the methanol concentration was ramped to 100% over 15 min. Unreacted ABOPX eluted in 11–12 min; [^125^I-]ABOPX eluted at 18–19 min in a yield of 50–60% of the initial ^125^I. In equilibrium binding assays the ratio of [^127^I/^125^I-]ABOPX was 10–20/1. Radioligand binding experiments were performed in triplicate with 20–25 μg membrane protein in a total volume of 0.1 mL HE buffer supplemented with 1 U/mL adenosine deaminase and 5 mM MgCl_2_. The incubation time was 3 h at 21°C. Nonspecific binding was measured in the presence of 100 mM NECA. Competition experiments were carried out using 0.6 nM ^125^I-ABOPX. Membranes were filtered on Whatman GF/C filters using a Brandell cell harvester (Gaithersburg, MD) and washed three times over 15–20 sec with ice-cold buffer (10 mM Tris, 1 mM MgCl_2_, pH 7.4). B_max_ and K_D_ values were calculated by Marquardt’s nonlinear least squares interpolation for single site binding models [Marquardt, 1963]. K_i_ values for different compounds were derived from IC_50_ values as described previously [Linden, 1982]. Data from replicate experiments are tabulated as means ± SEM.

[^3^H]CPX, ^125^I-ZM 241385 and ^125^I-ABA were utilized in radioligand binding assays to membranes derived from HEK-293 cells expressing recombinant human A_1_, A_2A_, and A_3_ adenosine receptors, respectively. Binding of [^3^H]*R*-*N*^6^-phenylisopropyladenosine ([^3^H]*R*-PIA; Amersham, Chicago, IL) to A_1_ receptors from rat cerebral cortical membranes and of [^3^H]CGS 21680 (NEN Life Sciences, Boston, MA) to A_2A_ receptors from rat striatal membranes was performed as described previously [Schwabe and Trost, 1980; Jarvis et al., 1989]. Adenosine deaminase (3 units/mL) was present during the preparation of the brain membranes in a preincubation of 30 min at 30°C and during the incubation with the radioligands. All nonradioactive compounds were initially dissolved in DMSO and diluted with buffer to the final concentration, where the amount of DMSO never exceeded 2%. Incubations were terminated by rapid filtration over Whatman GF/B filters using a Brandell cell harvester. The tubes were rinsed three times with 3 mL buffer each.

At least six different concentrations of competitor, spanning 3 orders of magnitude adjusted appropriately for the IC_50_ value of each compound, were used. IC_50_ values, calculated with the nonlinear regression method implemented in Graph-Pad Prism (San Diego, CA) were converted to apparent K_i_ values as described by Linden [1982]. Hill coefficients of the tested compounds were in the range of 0.8–1.1.

## RESULTS AND DISCUSSION

**Fig. 2.**
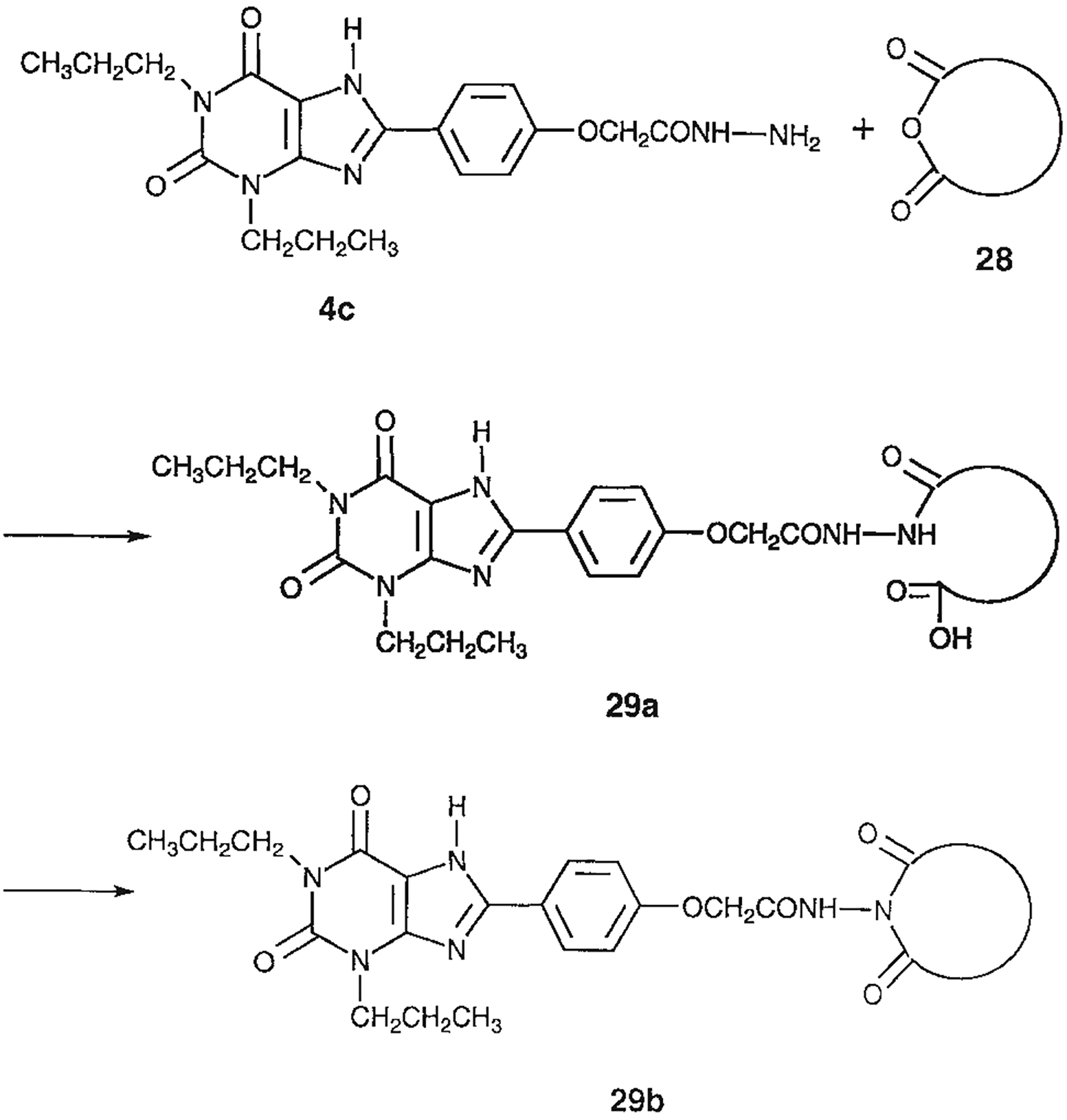
Derivatization of a xanthine containing a hydrazide group attached through the *p*-position of an 8-phenyl substituent [Jacobson et al., 1985]. The hydrazide, **4c**, was acylated with the anhydride, **28**, of a variety of dicarboxylic acids, followed by ring closure leading to stable imide derivatives, **29b**.

**Fig. 3.**
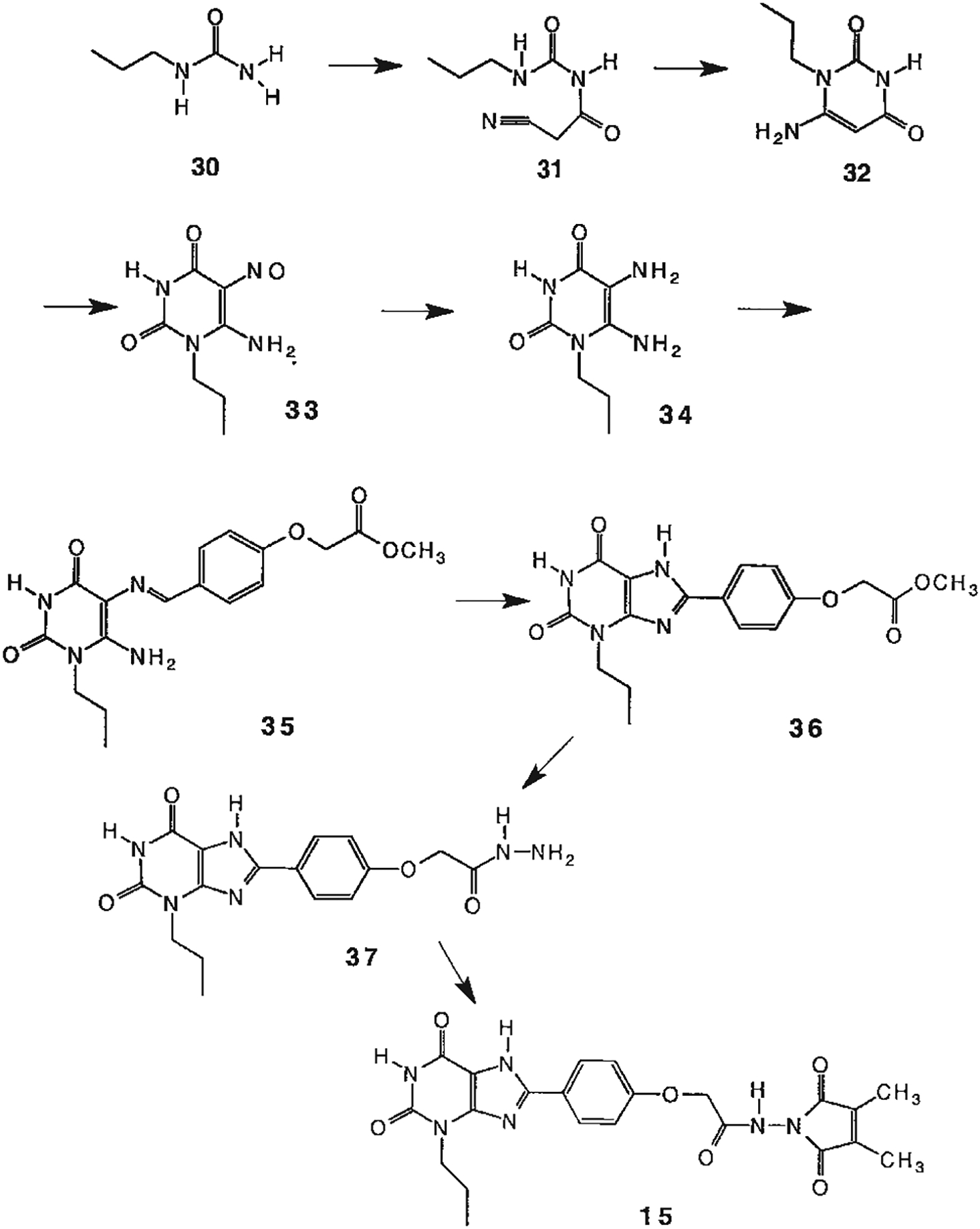
Synthesis of xanthine derivatives containing both 8-phenyl substituents and the 1-H-3-propyl substitution present in enprofylline, **1**, as potentially selective A_2B_ receptor antagonists.

The structures of the xanthine derivatives, **4**, **9–27**, tested for affinity in radioligand binding assays at adenosine receptors, are shown in Table 1. Most of the xanthines are derivatives of XCC [Jacobson et al., 1985], in which an acyl-hydrazide group is present. This group was included based on the high potency in the A_2B_ receptor binding assay (K_i_ value of 9.75 nM [Jacobson et al., 1999]) of an N-hydroxysuccinimide ester of XCC, **4d**. The hydrazide of XCC, **4c**, was acylated with a variety of mono- and dicarboxylic acids. Cyclization reactions were carried out for dicarboxylic acids, in two steps using the anhydride, **28**, for acylation, leading to imide (5- or 6-membered ring) derivatives (Fig. 2). The final step of ring-closure of **29a** to **29b** was effected at 50 °C, using excess carbodiimide and 1-hydroxybenzotriazole as catalyst. In some cases, where symmetric dicarboxylic acids were used, it was possible to isolate both the open structure, **29a**, and the cyclized imide form, **29b**. Pairs of open and cyclized derivatives of symmetric dicarboxylic acids prepared include compounds **18**–**20**. Also, the glutamic acid derivative **24a** was prepared using orthogonal protecting and the corresponding imide, **24b**. An 8-phenyl analog, **15**, of enprofylline was synthesized by standard methods from the asymmetric urea, **30** (Fig. 3).

At A_2B_ receptors, two radioligand binding assays (Table 1) were used. K_i_ values of xanthine derivatives were determined in displacement of binding of the non-selective radioligands [^3^H]ZM 241385, **8** (4-(2-[7-amino-2-{furyl}{1,2,4}triazolo{2,3-a}{1,3,5}tr nzyl)-8-phenyloxyacetate-1-propyl-xanthine), at human A_2B_ receptors expressed in HEK-293 cell membranes [Linden and Jacobson, 1998]. In order to evaluate selectivity, selected derivatives were subjected to standard binding assays at A_1_, A_2A_, and A_3_ receptors. The initial screening utilized rat brain A_1_/A_2A_ receptors (with radioligands [^3^H]*R*-PIA and [^3^H]CGS-21680), and selected compounds were examined at the recombinant human subtypes (Table 1), using [^3^H]CPX ([^3^H]8-cyclopentyl-1,3-dipropylxanthine) and ^125^I-ZM 241385, ^125^I-4-(2-[7-amino-2-[2-furyl][1,2,4]triazolo[2,3-*a*][1,3,5]triazin-5-yl-amino]ethyl)phenol) [Palmer et al., 1996]. Affinity at cloned human A_3_ receptors expressed in HEK-293 cells was determined using ^125^I-ABA (*N*^6^-(4-amino-3-[^125^I]iodobenzyl)-adenosine) and ^125^I-AB-MECA (*N*^6^-(4-amino-3-iodobenzyl)-adenosine-5′-*N*-methyluronamide).

The initial screening utilized rat A_1_/A_2A_ receptors, and selected compounds were examined at the human subtypes. Selectivities for the human A_2B_ vs. rat A_1_/A_2A_ receptors were generally small (3–4-fold at best), while comparisons within the same species (human) generally lead to greater selectivities. A 1,2-dimethylmaleimide derivative, **14**, bound to human A_2B_ receptors with a K_i_ of 19 nM and proved to be selective vs. human A_1_/A_2A_/A_3_ receptors by 160-, 100-, and 35-fold, respectively.

Enprofylline (3-propylxanthine) is slightly selective for A_2B_ receptors; however, combination of the 1-H-3-Pr and 8-phenyl substituents eliminated the selectivity (cf. **14** and **15**).

Other potent and moderately selective A_2B_ antagonists were a tetrahydrophthaloyl derivative **18b** (K_i_ value 10 nM) and amino acid conjugates of the XCC-hydrazide, i.e. the glutarimide **24b** (K_i_ value 13 nM) and protected dipeptide **27** (K_i_ value 11 nM). Compound **20a** displayed a K_i_ value of 17 nM. Other derivatives displaying selectivity for A_2B_ receptors, but with less potency (K_i_ values in nM in parentheses) were: **11** (30), **16** (67), **17** (28), **24a** (25), **25** (48), and **26** (40). A direct comparison of either shows increased (**18b** or **19b**) or decreased (**20b**) A_2B_ receptor affinity upon cyclization.

The identification of **14** (MRS 1595) as an adenosine antagonist which is potent and selective for human A_2B_ receptors and should be hydrolytically stable will provide an opportunity to test the hypothesis that this subtype is involved in asthma. Further SAR studies are in progress to enhance the pharmacological profile of these xanthine derivatives as A_2B_ receptor antagonists.

## Figures and Tables

**Fig. 1.**
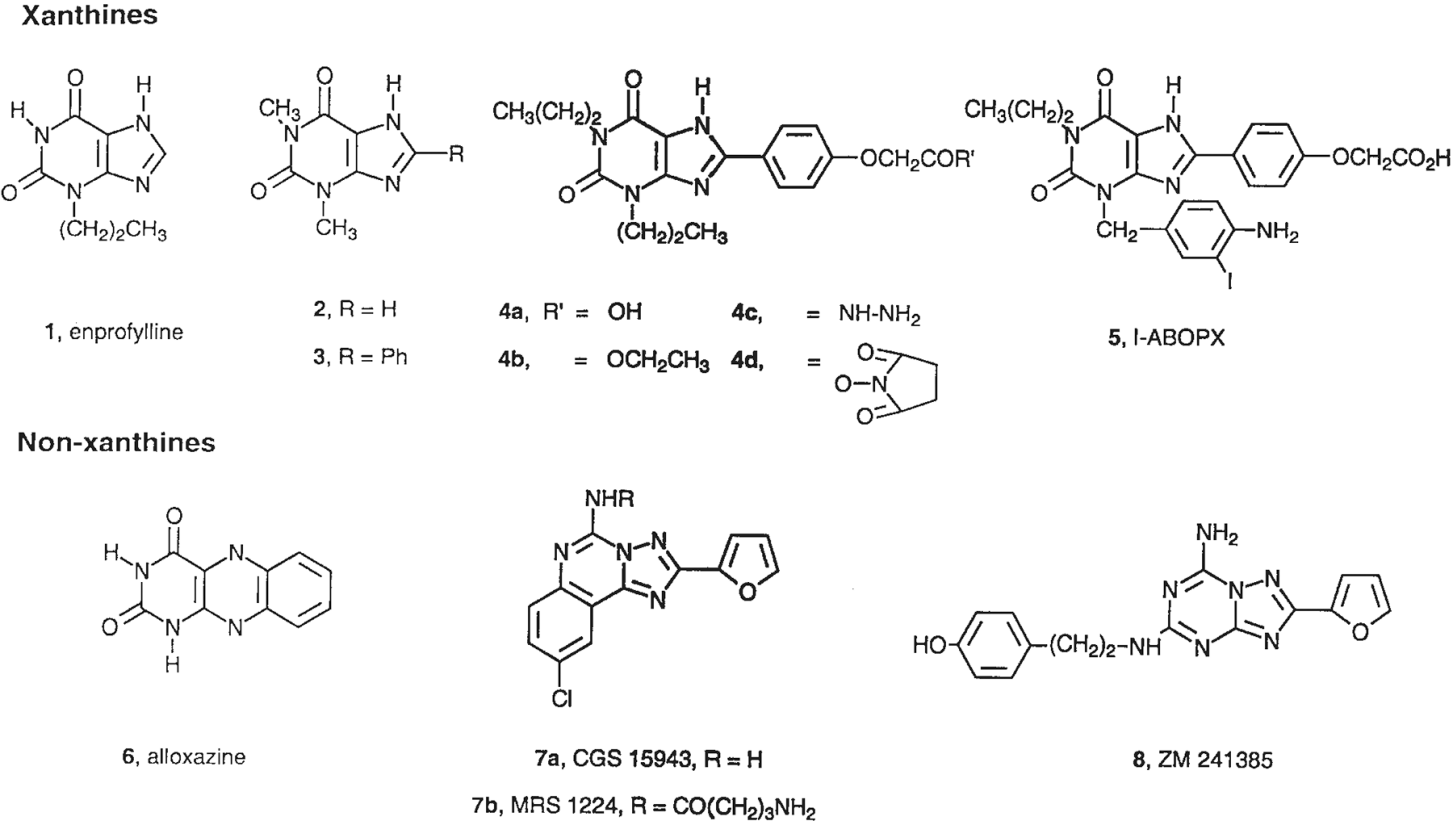
Structures of xanthines and nonxanthines previously identified as antagonists at A_2B_ receptors.

**TABLE 1.** Affinities of Xanthine Derivatives in Radioligand Bindng Assays at Rat A_1_,^a^ Rat A_2B_,^b^ Human A_2B_,^b^ and Human A_3_ receptors,^c^ unless noted.^e^

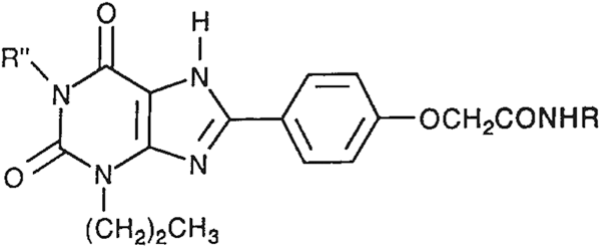							
			Ki (nM) or % displacement				
Compund	R	R″	rA_1_^a^	rA_2A_^b^	hA_2B_^b^	hA_3_^c^	rA_1_/hA_2B_
4b	—	Pr	51.6 ± 8.0, 203 ± 59(h)^e^	128 ± 15, 342 ± 10(h)^e^	18.7 ± 0.5, 34.5 ± 6.3^e^	48.5 ± 0.8^e^	2.8
4c	NH_2_	Pr	16.0 ± 0.5	63.8 ± 21.3	13.2 ± 5.9	498 ± 139	1.2
4e	NH-COCH_3_	Pr	6.51 ± 1.24, 125 ± 14(h)^e^	227 ± 64, 186 ± 9(h)^e^	65.4 ± 6.5, 33.8 ± 13.7^e^	30.9 ± 8.2^e^	0.10
9	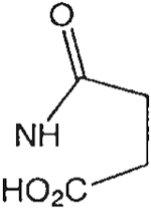	Pr	73.3 ± 22.0, 219 ± 3(h)^e^	174 ± 32, 795 ± 98(h)^e^	116 ± 10, 97.8 ± 3.3^e^	173 ± 27^e^	1.6
10	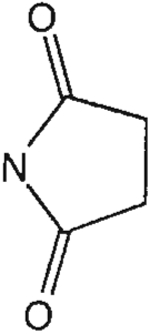	Pr	55.9 ±25.1 75.2 ± 5.5(h)^e^	805 ± 44 27.2 ± 8.6 (h)^e^	18.6 ± 6.1	766 ± 176	3.0
11	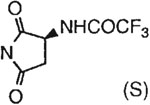	Pr	74.3 ± 6.6	139 ± 32	30.2 ± 0.5	1,560	2.5
12	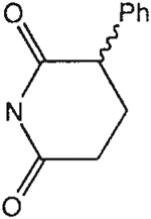	Pr	3.87 ± 1.20	21.4 ± 6.1	3.86 ± 0.7	151 ±99	1.0
13	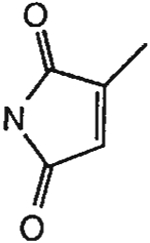	Pr	203 ± 41	1,230 ± 270	144 ± 11	551 ± 106	1.4
14^d^	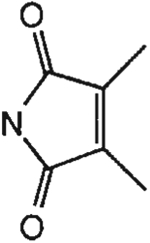	Pr	11.1 ± 2.4, 3,030 ± 1110 (h)^e^	126 ± 41, 1,970 ± 550 (h)^e^	19.4 ± 6.2, 33.8 ± 1.9^e^	670 ± 154^e^	0.57
15	″	H	3,590 ±920, 8,080 ± 1720 (h)^e^	36 ± 4% (10^−4^) 5,480 ± 920 (h)^e^	1,800 ± 0, 1,900 ± 280^e^	14,200 ± 11,500^e^	2.0
16	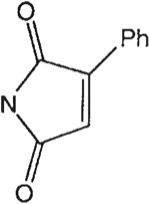	Pr	225 ± 76	1,540 ± 280	66.7 ± 37.0	748 ± 234	3.4
17	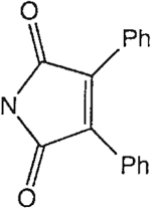	Pr	95.8 ± 25.1	2,100 ± 630	27.9 ± 8.5	3,450 ± 1,470	3.4
18a	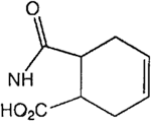	Pr	134 ± 19	813 ± 299	51.0 ± 7.0	1,060 ± 150	2.6
18b	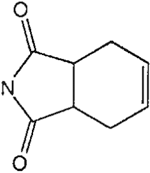	Pr	36.4 ± 6.2 129 ± 20 (h)^e^	689 ± 477 301 ± 31 (h)^e^	10.0 ± 3.0	370 ± 190	3.6
19a	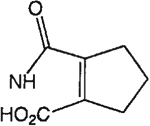	Pr	81.7 ± 31.2	708 ± 169	78.5 ± 20.5	1,180 ± 700	1.0
19b	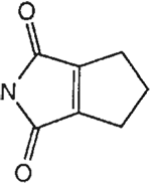	Pr	41.3 ± 6.4	1,160 ± 337	21.5 ± 1.5	308 ± 88	1.9
20a	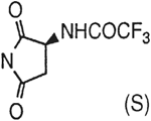	Pr	47.2 ± 6.8 145 ± 11 (h)^e^	422 ± 136 95.6 ± 16.8 (h)^e^	17.3 ± 6.3	438 ± 109	2.7
20b	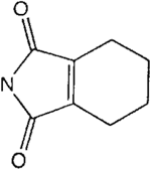	Pr	61.9 ± 11.3	415 ± 157	35.8 ± 0.7	245 ± 45	1.7
21	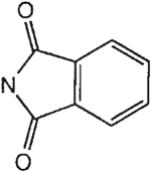	Pr	26.3 ± 2.3, 210 ± 42 (h)^e^	392 ± 117, 359 ± 21 (h)^e^	64.4 ± 0.8, 46.4 ± 14.5^e^	147 ± 21^e^	0.41
22	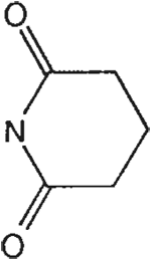	Pr	14.0 ± 2.3	135 ± 39	22.0 ± 5.5	200 ± 45	0.6
23	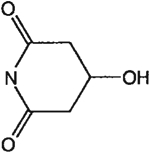	Pr	41.2 ± 16.6	164 ± 61	25.7 ± 5.5	290 ± 88	1.6
24a	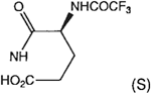	Pr	70.8 ± 30.9	872 ± 412	24.8 ± 7.3	430 ± 44	2.9
24b	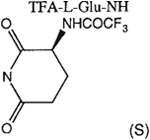	Pr	53.5 ± 6.5 149 ± 6 (h)^e^	440 ± 106 178 ± 20 (h)^e^	13.0 ± 3.5	726 ± 245	4.1
25	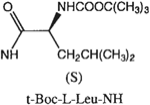	Pr	197 ± 67	2,750 ± 950	47.5 ± 2.5	195 ± 84	4.1
26	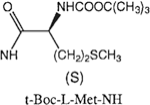	Pr	113 ± 27	524 ± 285	39.7 ± 13.6	690 ±570	2.8
27	**Cbz-Gly_2_-NH**	Pr	36.0 ± 6.6 200 ± 22 (h)^e^	609 ± 95 830 ± 84 (h)^e^	10.8 ± 5.0	323 ± 47	3.3

a Displacement of specific [^3^H]*R* - PIA binding to A_1_ receptors in rat brain membranes, expressed as K_i_ ± S.E.M. (n = 3–5), unless noted.

b Displacement of specific [^3^H]CGS 21680 binding to A_2A_ receptors in rat striatal membranes, expressed as K_i_ ± S.E.M. (n = 3–6), and at A_2B_ receptors expressed in HEK-293 cells vs [^3^H]ZM241385, unless noted.

c Displacement of specific [^125^I]AB-MECA binding at human A_3_ receptors expressed in HEK cells, in membranes, expressed as K_i_ ± S.E.M. (n = 3–4), unless noted.

d MRS 1595.

e K_i_ values were determined in radioligand binding assays at recombinant human A_1_ and A_2A_ receptors expressed inHEK-293 cells vs [^3^H]CPX and [^125^I]ZM241385, respectively. Affinity of xanthine derivatives at human A_2B_ receptors expressed in HEK-293 cells was determined using [^125^I]-ABOPX. Affinity at recombinant human A_3_ receptors expressed in HEK-293 cells was determined using [^125^I]ABA.

## Abbreviations:

CGS: 21680 2-[4-[(2-carboxyethyl)phenyl]ethylamino]-5′-*N*-ethylcarbamoyl adenosine
CHA: *N*^6^-cyclohexyladenosine
CHO: Chinese hamster ovary cells
CPX: 8-cyclopentyl-1,3-dipropylxanthine
DIPEA: diisopropylethylamine
DMAP: 4-dimethylaminopyridine
DMF: N,N-dimethylformamide
DMSO: dimethylsulfoxide
EDTA: ethylenediaminetetraacatate
HEK cells: human embryonic kidney cells
HOBt: 1-hydroxybenzotriazole
[^125^I]ABA: [^125^I]*N*^6^-(4-aminobenzyl)-adenosine
[^125^I]AB-MECA: [^125^I]*N*^6^-(4-amino-3-iodobenzyl)-adenosine-5′-*N*-methyluronamide
^125^I-ABOPX: ^125^I-3-(4-amino-3-iodobenzyl)-8-oxyacetate-1-propylxanthine
K_i_: equilibrium inhibition constant
NECA: 5′-(*N*-ethylcarbamoyl)adenosine
NHS: N-hydroxysuccinimide ester
*R*-PIA: *R*-*N*^6^-phenylisopropyladenosine
SAR: structure–activity relationship
TFA: trifluoroacetic acid
TFAA: trifluoroacetic anhydride
Tris: tris(hydroxymethyl)aminomethane
XAC: 8-[4-[[[[(2-aminoethyl)-amino]carbonyl]methyl]oxy]phenyl]-1,3-dipropylxanthine
XCC: 8-[4-[[[carboxy]methyl]oxy]phenyl]-1,3-dipropylxanthine
